# Lower-Limb Joint Work Redistribution and Altered Movement Control During Double-Leg Squatting in Patients 6 Months After Anterior Cruciate Ligament Reconstruction

**DOI:** 10.3390/bioengineering13091080

**Published:** 2026-09-18

**Authors:** Xinyue Liu, Huijuan Shi, Xi Gong, Huimeng Chen, Yujiao Wang, Xiaohan Zhao, Shuang Ren, Hui Liu

**Affiliations:** 1 Key Laboratory of Exercise Rehabilitation Science of Ministry of Education, College of Human Movement Science, Beijing Sport University, Beijing100084, China; 13931378102@163.com (X.L.); shihuijuan1103@163.com (H.S.); chenhuimeng@bsu.edu.cn (H.C.); 2 Department of Sports Medicine, Institute of Sports Medicine, Peking University, Peking University Third Hospital, Beijing 100191, China; gongxi518@163.com (X.G.); 15806802061@163.com (Y.W.); a15963957860@163.com (X.Z.); 3 Beijing Key Laboratory of Research and Translation for Drugs and Medical Devices in Precision Diagnosis and Treatment of Sports Injuries, Beijing 100191, China; 4 Engineering Research Center of Sports Trauma Treatment Technology and Devices, Ministry of Education, Beijing 100191, China; 5 School of Kinesiology and Health, Capital University of Physical Education and Sports, Beijing 100191, China

**Keywords:** double-leg squatting, anterior cruciate ligament reconstruction, lower-limb biomechanics, compensatory strategy, movement control

## Abstract

Individuals undergoing anterior cruciate ligament reconstruction (ACLR) remain at risk of secondary injury and joint degeneration, highlighting the need for functional assessments that detect abnormal load regulation and compensatory movement strategies. This study compared lower-limb kinematics, kinetics, and joint work contributions during double-leg squatting between 27 patients 5.9 ± 0.7 months after ACLR and 23 healthy controls. Three-dimensional marker trajectories and ground reaction forces were collected, and biomechanical variables were analyzed using a two-way mixed-design ANOVA. The reconstructed limb showed smaller knee flexion angles and lower knee extensor moments than both the contralateral limb and the non-dominant limb of healthy controls (*p* ≤ 0.015), together with reduced ankle dorsiflexion and peak vertical ground reaction force versus the contralateral limb (*p* < 0.001). Compared with healthy controls, the ACLR group also showed altered frontal- and transverse-plane hip and knee mechanics (*p* ≤ 0.017). The reconstructed limb demonstrated greater negative hip work and hip work contribution, lower negative knee work and knee work contribution (all *p* ≤ 0.003), and greater ankle work contribution than the contralateral limb (*p* = 0.018). These findings describe a cross-sectional biomechanical pattern at approximately 6 months after ACLR characterized by sagittal-plane knee control deficits, bilateral hip control differences, and altered inter-joint work distribution.

## 1. Introduction

Anterior cruciate ligament (ACL) rupture is one of the most common musculoskeletal injuries in physically active populations [1,2]. Although anterior cruciate ligament reconstruction (ACLR) can restore mechanical stability of the knee joint [3], postoperative functional recovery remains suboptimal. Previous studies have shown that, compared with healthy controls or the contralateral limb, the reconstructed limb of patients after ACLR still shows quadriceps weakness [4], reduced limb loading [5], and neuromuscular control deficits [6]. These postoperative functional deficits not only hinder the return to sports but also increase the risk of knee osteoarthritis and secondary ACL injury [7,8]. Therefore, precise functional assessment is crucial for identifying residual impairments and optimizing targeted rehabilitation.

Current functional assessments after ACLR have limitations in both task selection and the choice of reference standards. Postoperative functional assessments mainly focus on demanding and high-impact tasks, such as running [9,10], jump landing [11], single-leg hop tests [12], and change-of-direction tasks [13]. These studies have shown that patients after ACLR demonstrate movement patterns that differ from those of healthy controls at 1 year postoperatively or at the time of return to sport. These patterns include a smaller knee flexion range of motion, a lower knee extensor moment, and reduced knee joint work [14,15,16,17]. In addition, reduced knee extensor moment symmetry during jump landing, increased knee valgus range of motion in the reconstructed limb [7], and poor limb symmetry during single-leg hop tests [18] may reflect insufficient postoperative functional recovery. These deficits are also associated with an increased risk of secondary ACL injury. Although these tasks are valuable for identifying high-risk movement patterns, they require substantial physical capacity that many patients have not yet achieved at 6 months after surgery. This limitation may lead to incomplete functional screening during the critical early-to-mid postoperative phase. In addition, previous clinical assessments often use the contralateral limb as the normal reference. However, evidence suggests that the contralateral limb may also show neuromuscular abnormalities after ACLR, especially reduced explosive force production [19]. This indicates that biomechanical abnormalities after ACLR are not limited to the reconstructed limb. Therefore, assessing postoperative recovery based only on one limb or on limb symmetry indices may underestimate the true functional deficits of patients after ACLR. Thus, a clinically feasible low-load assessment that can identify residual movement deficits and compensatory strategies, rather than relying solely on task performance or interlimb symmetry, is still needed during the early-to-mid stage after ACLR.

Compared with the high-load tasks described above, double-leg squatting is a relatively low-load, safe, and easily standardized functional task that is clinically feasible for repeated testing [20,21]. Given its biomechanical relationship with high-impact landing tasks [22,23], double-leg squatting serves as a low-load alternative screening task to assess lower-limb movement control patterns after ACLR. Prior studies have identified interlimb asymmetries in lower-limb joint loading [24,25] and kinematics [26,27] during squatting, as well as a shift in support moment from the knee toward the ankle or hip within the reconstructed limb [28]. However, most previous biomechanical studies of squatting have focused on peak angles, peak moments, or support moments at specific time points within a single joint or plane [28,29,30,31]. Such approaches may fail to capture the continuous distribution of load across joints throughout the entire movement. In contrast, joint work and joint work contribution can provide a more comprehensive description of energy distribution and movement control strategies throughout the task [32]. Previous evidence has shown that patients after ACLR often demonstrate increased hip work and reduced knee work during single-leg vertical jumping [17]. However, there is still limited evidence on whether a similar work redistribution pattern occurs during the lower-load double-leg squat, particularly at approximately 6 months after ACLR, when patients may be able to complete the task despite persistent functional deficits. Clarifying this redistribution is clinically relevant because successful task completion may mask persistent knee unloading when mechanical demand is redistributed to the hip or ankle.

To address these research and clinical gaps, the present study evaluated lower-limb kinematics, kinetics, joint work, and joint work contribution during double-leg squatting in patients 6 months after ACLR and healthy controls. By comparing both limbs of patients with healthy reference limbs, this study aimed to identify residual movement control deficits and inter-joint work redistribution that may inform postoperative functional assessment and rehabilitation. We hypothesized that: (1) the reconstructed limb would show smaller knee flexion and ankle dorsiflexion angles, as well as a lower knee extensor moment during double-leg squatting compared with the contralateral limb and healthy controls; (2) the reconstructed limb would show greater hip and ankle work contribution, but lower knee work contribution during double-leg squatting compared with the contralateral limb and healthy controls.

## 2. Materials and Methods

### 2.1. Participants

A total of 32 patients who had undergone unilateral ACLR with an autologous hamstring tendon graft were recruited for the ACLR group. Twenty-three healthy participants were recruited for the control group. The inclusion criteria for the ACLR group were as follows: adults aged 18–45 years [33]; patients who had undergone primary unilateral ACLR; no severe ankle sprain within the previous 6 months; no other musculoskeletal or neurological conditions unrelated to the index knee injury that would preclude safe completion of the testing protocol; and willingness to participate and provide written informed consent. The exclusion criteria were revision or multiple ACLR procedures, a high risk of postoperative infection, or other postoperative complications. The inclusion criteria for the control group were age 18–45 years, a Tegner activity level of ≥6, and no signs or symptoms of musculoskeletal disorders within the previous 6 months. Healthy controls were recruited with the aim of achieving group-level comparability with the ACLR cohort in age, sex, height, body mass, BMI, and activity level. Activity-level comparability was evaluated relative to the preoperative Tegner level of the ACLR group. No individual one-to-one matching procedure was used. The exclusion criteria were a history of ACL injury or any disease that limited exercise testing. All inclusion and exclusion criteria were confirmed by two researchers.

Sample size was estimated using G*Power version 3.1.9.2 (Heinrich Heine University Düsseldorf, Düsseldorf, Germany). Based on a previous study [28], the partial eta squared for peak knee extensor moment was estimated as 0.12, corresponding to an effect size of 0.37. The statistical test was set as F tests, ANOVA: repeated measures, within–between interaction. Power was set at 80%, and the alpha level was set at 0.05. The number of groups was set as 2, and the number of measurements was set as 2. The minimum required sample size was 18 participants. Five participants in the ACLR group did not meet the eligibility criteria. Therefore, 27 eligible patients after ACLR were included in the final analysis. Among them, 12 had undergone left-side reconstruction and 15 had undergone right-side reconstruction. Available clinical records showed that 14 of the 27 patients (51.9%) had concomitant meniscal injury, of whom 7 (25.9% of the total cohort) underwent meniscal repair. No concomitant medial or lateral collateral ligament injury was recorded. Chondral debridement was recorded in 9 patients (33.3%). Twenty-three healthy participants were included in the control group. Among the controls, 4 had a left dominant limb and 19 had a right dominant limb. No significant differences in demographic characteristics were found between groups (*p* ≥ 0.053) (Table 1).

### 2.2. Data Collection

Before formal testing, participants completed a 5 min warm-up. They were then informed of the testing procedures, familiarized with the test movements, and asked to sign the informed consent form. All participants wore standardized tight-fitting clothing and sports shoes. A total of 33 reflective markers were placed on anatomical landmarks. These landmarks included the bilateral acromion processes, anterior superior iliac spines, posterior superior iliac spines, highest points of the iliac crests, anterior thighs, lateral thighs, lateral femoral epicondyles, medial femoral epicondyles, tibial tuberosities, lateral shanks, lateral malleoli, medial malleoli, heels, fifth metatarsophalangeal joints, second metatarsophalangeal joints, first metatarsophalangeal joints, and one marker over the lumbar region.

An eight-camera infrared motion capture system was used to record marker trajectories at 200 Hz (Qualisys, Gothenburg, Sweden). Ground reaction forces during squatting were recorded synchronously at 1000 Hz using three-dimensional force plates (Kistler, Winterthur, Switzerland). Each participant first completed a static standing calibration trial. They then performed five consecutive double-leg squats at a self-selected speed. Participants were instructed to keep their hands on their hips, place their feet shoulder-width apart, and stand with each foot on a separate force plate. Both toes pointed forward. Participants squatted until their thighs were parallel to the ground and then returned to the standing position [23]. All participants included in the final analysis were able to complete the standardized double-leg squat protocol, and no pain or adverse symptoms requiring modification or termination of the test were reported. However, formal clinical clearance for unrestricted squatting or sports participation was not systematically documented.

### 2.3. Data Processing

Data analysis was performed using Visual3D (HAS-Motion, Kingston, ON, Canada) and MATLAB R2024a (The MathWorks, Natick, MA, USA). Marker trajectory data and ground reaction force data were filtered using a fourth-order Butterworth low-pass filter. The cut-off frequencies were 13.3 Hz [34] and 200 Hz, respectively. The pelvis was defined using the CODA model. The hip joint center was determined according to Bell et al. [35]. The knee joint center was defined as the midpoint between the medial and lateral femoral epicondyles. The ankle joint center was defined as the midpoint between the medial and lateral malleoli. Hip, knee, and ankle joint angles were calculated using Cardan angles with an X–Y–Z rotation sequence [36]. Lower-limb joint moments were calculated using inverse dynamics and normalized to body mass (Nm/kg). Flexion moments were defined as positive values. Ground reaction forces were normalized to body mass (N/kg).

The eccentric phase of the squat was defined as the period from squat initiation, identified when the vertical velocity of the pelvis exceeded 5 cm/s, to the lowest position of the pelvic center of mass [5]. During the eccentric phase of each squat, peak hip, knee, and ankle joint angles, peak joint moments, and peak ground reaction forces were calculated. Joint negative work was calculated by integrating the joint power–time curve during the eccentric phase. Joint work contribution was then calculated for each joint. Hip work contribution (%) was defined as the percentage of hip joint work relative to the total work of the hip, knee, and ankle joints during the eccentric phase. Knee and ankle work contributions were calculated in the same way. In addition, key variables were extracted at peak knee extensor moment during the eccentric phase of the squat. These variables included trunk angles, as well as hip, knee, and ankle joint angles and joint moments.

### 2.4. Statistical Analysis

Data normality was assessed using the Shapiro–Wilk test and Q–Q plots. A two-way mixed-design analysis of variance was used to examine the effects of group and limb on biomechanical variables during the double-leg squat. The group factor included patients after ACLR and healthy controls. The limb factor included the reconstructed and contralateral limbs in patients after ACLR, and the dominant and non-dominant limbs in healthy controls. The dominant limb was defined as the limb that the participant usually used to kick a ball. When a significant group-by-limb interaction was detected, simple effects analyses were performed. When no interaction effect was detected, the main effects were reported. Bonferroni correction was used for post hoc comparisons. Effect sizes for interaction effects and main effects were reported as partial eta squared. For simple-effects comparisons, within-participant interlimb comparisons were quantified using Hedges-corrected paired standardized mean differences (*g_z_*), whereas between-group limb comparisons were quantified using Hedges’ g (*g*) for independent samples. Standardized mean-difference effect sizes were reported as absolute values, with the direction of differences determined from the descriptive statistics. The level of statistical significance was set at *p* < 0.05. All statistical analyses were performed using SPSS version 29.0.

## 3. Results

### 3.1. Trunk Angle and Lower-Limb Joint Angles and Moments at Peak Knee Extensor Moment

Significant group-by-limb interactions were observed for knee flexion and ankle dorsiflexion angles (Table 2). In patients after ACLR, the reconstructed limb showed less knee flexion (*p* = 0.003, *g_z_* = 0.563) and ankle dorsiflexion (*p* < 0.001, *g_z_* = 0.836) than the contralateral limb. No significant interlimb differences were observed in healthy controls (*p* ≥ 0.169). The reconstructed limb also showed less knee flexion than the non-dominant limb of healthy controls (*p* = 0.004, *g* = 0.846), while the contralateral limb did not differ from the dominant limb of healthy controls (*p* = 0.321, *g* = 0.280). No significant group-by-limb interactions were observed for hip abduction or ankle internal rotation angles. Hip abduction angle was smaller in the ACLR group than in healthy controls (*p* = 0.014, *η*^2^ = 0.118), with a significant main effect of limb (*p* = 0.033, *η*^2^ = 0.091). Ankle internal rotation angle showed a significant main effect of limb (*p* = 0.015, *η*^2^ = 0.116), but no significant group effect (*p* = 0.137, *η*^2^ = 0.045). No significant group or limb effects were found for trunk angle, hip flexion or internal rotation angles, knee adduction or internal rotation angles, or ankle inversion angle (group: *p* ≥ 0.119; limb: *p* ≥ 0.051).

A significant group-by-limb interaction was observed for knee extensor moment (Table 3). The reconstructed limb of the ACLR group showed a lower knee extensor moment than both the contralateral limb (*p* < 0.001, *g_z_* = 1.220) and the non-dominant limb of healthy controls (*p* = 0.002, *g* = 0.909). No interlimb difference was observed in healthy controls (*p* = 0.946, *g_z_* = 0.014), and the contralateral limb of the ACLR group did not differ from the dominant limb of healthy controls (*p* = 0.908, *g* = 0.032). No group-by-limb interactions were observed for hip adduction, knee external rotation, or ankle internal/external rotation moments. Significant group effects were found for all three variables. Compared with healthy controls, the ACLR group exhibited lower hip adduction (*p* = 0.001, *η*^2^ = 0.192) and knee external rotation moments (*p* = 0.003, *η*^2^ = 0.164). A significant group effect was also observed for ankle internal/external rotation moment (*p* = 0.027, *η*^2^ = 0.097). No limb effects were found for these variables (*p* ≥ 0.125). No significant group or limb effects were found for hip extensor or internal rotation moments, knee abduction moment, ankle plantarflexion moment, or ankle eversion moment (group: *p* ≥ 0.219; limb: *p* ≥ 0.092).

### 3.2. Peak Lower-Limb Joint Angles and Moments During the Eccentric Phase of the Squat

Significant group-by-limb interactions were observed for peak knee flexion and ankle dorsiflexion angles (Table 4). In patients after ACLR, the reconstructed limb had a smaller peak knee flexion (*p* = 0.003, *g_z_* = 0.603) and ankle dorsiflexion angle (*p* < 0.001, *g_z_* = 0.817) than the contralateral limb. No significant interlimb differences were observed in healthy controls (*p* ≥ 0.093). The reconstructed limb also showed a smaller peak knee flexion angle than the non-dominant limb of healthy controls (*p* = 0.015, *g* = 0.707), whereas no significant differences were observed between the contralateral limb and the dominant limb of healthy controls (*p* ≥ 0.132). No significant group-by-limb interaction was found for peak hip abduction angle. The ACLR group showed a smaller peak hip abduction angle than healthy controls (*p* = 0.017, *η*^2^ = 0.112). A significant limb effect was found across groups (*p* = 0.035, *η*^2^ = 0.089). No significant group or limb effects were found for peak hip flexion, hip internal rotation, knee adduction, ankle inversion, or ankle internal rotation angles (group: *p* ≥ 0.188; limb: *p* ≥ 0.067).

A significant group-by-limb interaction was found for peak knee extensor moment (Table 5). The reconstructed limb of the ACLR group showed a lower peak knee extensor moment than both the contralateral limb (*p* < 0.001, *g_z_* = 1.216) and the non-dominant limb of healthy controls (*p* = 0.002, *g* = 0.912). No interlimb difference was observed in healthy controls (*p* = 0.973, *g_z_* = 0.007), and the contralateral limb of the ACLR group did not differ from the dominant limb of healthy controls (*p* = 0.928, *g* = 0.025). No significant group-by-limb interactions were observed for peak hip adduction, knee external rotation, or ankle external rotation moments. Compared with healthy controls, the ACLR group exhibited lower peak hip adduction (*p* < 0.001, *η*^2^ = 0.208), knee external rotation (*p* = 0.003, *η*^2^ = 0.168), and ankle external rotation moments (*p* = 0.017, *η*^2^ = 0.113). No significant limb effects were found for these variables (*p* ≥ 0.123). No significant group or limb effects were found for peak hip extensor or internal rotation moments, knee abduction moment, ankle plantarflexion moment, or ankle eversion moment (group: *p* ≥ 0.295; limb: *p* ≥ 0.123).

### 3.3. Peak Vertical Ground Reaction Force During the Eccentric Phase of the Squat

A significant group-by-limb interaction was found for peak vertical ground reaction force (Table 6). In patients after ACLR, the reconstructed limb had a lower peak vertical ground reaction force than the contralateral limb (*p* < 0.001, *g_z_* = 0.838). No significant interlimb difference was observed in healthy controls (*p* = 0.206, *g_z_* = 0.344), and neither the reconstructed nor contralateral limb of the ACLR group differed from the corresponding limb of healthy controls (*p* ≥ 0.054).

### 3.4. Negative Lower-Limb Joint Work and Joint Work Contribution During the Eccentric Phase of the Squat

**Figure 1:**
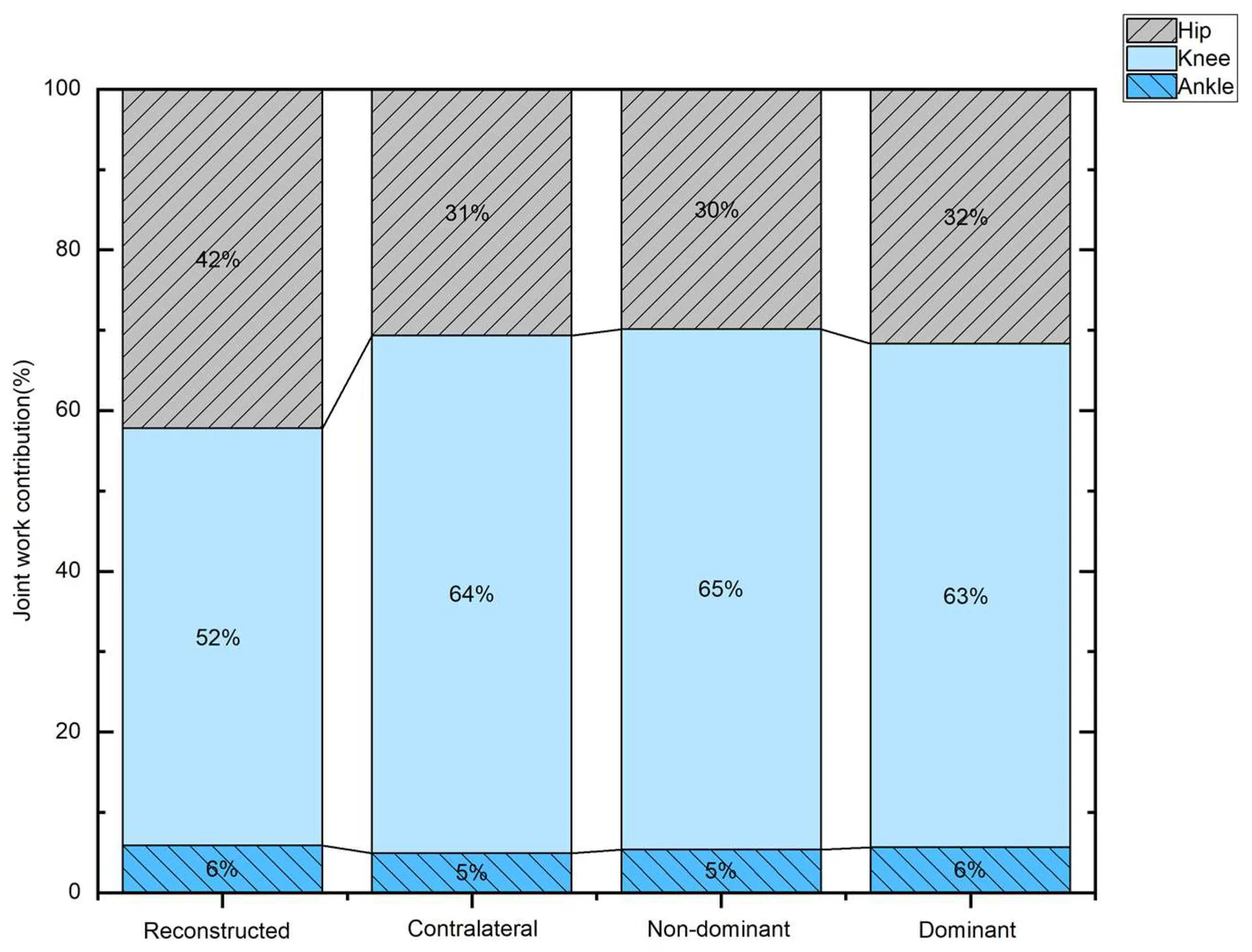
The work contribution of hip, knee, and ankle joints during eccentric phase of squat.

Significant group-by-limb interactions were observed for negative hip and knee work and for hip, knee, and ankle work contributions (Table 7, Figure 1). the reconstructed limb showed greater negative hip work (*p* < 0.001, *g_z_* = 0.880), hip work contribution (*p* < 0.001, *g_z_* = 1.391), and ankle work contribution (*p* = 0.018, *g_z_* = 0.489), but lower negative knee work (*p* < 0.001, *g_z_* = 1.501) and knee work contribution (*p* < 0.001, *g_z_* = 1.394), than the contralateral limb. No significant interlimb differences were observed in healthy controls (*p* ≥ 0.111). Compared with the non-dominant limb of healthy controls, the reconstructed limb showed greater negative hip work (*p* = 0.003, *g* = 0.874) and hip work contribution (*p* < 0.001, *g* = 1.230), but lower negative knee work (*p* < 0.001, *g* = 1.232) and knee work contribution (*p* < 0.001, *g* = 1.282). The contralateral limb did not differ from the dominant limb of healthy controls (*p* ≥ 0.555). No significant group or limb effects were found for negative ankle work (group: *p* = 0.538, *η*^2^ = 0.008; limb: *p* = 0.824, *η*^2^ = 0.001).

## 4. Discussion

The purpose of this study was to investigate alterations in lower-limb joint kinematics, kinetics, and joint work distribution during double-leg squatting in patients 6 months after ACLR. The main findings showed that, during double-leg squatting, the reconstructed limb exhibited a smaller knee flexion angle than the contralateral limb and healthy controls, a smaller ankle dorsiflexion angle than the contralateral limb, and a smaller hip abduction angle than healthy controls. The reconstructed limb also demonstrated a lower knee extensor moment than the contralateral limb and healthy controls, as well as lower ankle external rotation, knee external rotation, and hip adduction moments than healthy controls. Further analysis of joint work showed that the reconstructed limb exhibited greater negative hip work and hip work contribution than the contralateral limb and healthy controls, lower negative knee work and knee work contribution than the contralateral limb and healthy controls, and a greater ankle work contribution than the contralateral limb. These findings indicate that the biomechanical alterations observed during double-leg squatting in patients 6 months after ACLR were primarily characterized by impaired movement control in the sagittal plane of the knee and the frontal plane of the hip, together with altered energy distribution across the lower-limb joints.

The findings of this study partially supported the first hypothesis. The reconstructed limb exhibited a smaller knee flexion angle and lower knee extensor moment than the contralateral limb and healthy controls, a smaller ankle dorsiflexion angle than the contralateral limb, and a smaller hip abduction angle and lower hip adduction moment than healthy controls. These findings are consistent with a knee-protective movement pattern in the reconstructed limb during double-leg squatting. Similar patterns have been observed during single-leg squatting [37], double-leg squatting [28,38], running [39], and jumping tasks [40], primarily characterized by smaller peak knee flexion angles and lower knee extensor moments. Previous research has shown that greater quadriceps strength is associated with a greater peak knee flexion angle [41]. During squatting, a smaller peak knee flexion angle may reduce the demand placed on the quadriceps [41], thereby potentially limiting anterior tibial translation and reducing graft loading [42]. Similarly, a smaller ankle dorsiflexion angle may restrict forward displacement of the knee and reduce quadriceps demand, which is consistent with previous findings [27]. The kinetic findings of the present study further support the load-regulation strategy reflected by these kinematic alterations. The reconstructed limb showed lower knee extensor output during double-leg squatting, consistent with lower knee joint loading and a knee-protective movement pattern. When investigating movement control strategies during double-leg squatting, Paulien et al. [28] reported that although the total support moment was similarly distributed between the involved and uninvolved limbs after ACLR, the contribution of the knee was reduced, whereas the contributions of the ankle and hip were increased. This multi-joint compensatory strategy may protect the knee joint and is consistent with the findings of the present study. Collectively, patients after ACLR may complete the double-leg squat by coordinately adjusting sagittal-plane joint angles and moment distribution to limit anterior knee displacement and reduce the demand placed on the knee extensors.

The present study further found that the ACLR group exhibited a smaller hip abduction angle and lower hip adduction moment than the healthy control group. Unlike the kinematic and kinetic alterations observed at the knee of the reconstructed limb, these variables showed no significant interaction effects and were primarily characterized by main effects of group. This finding suggests that the frontal-plane alterations at the hip may not represent a localized difference specific to the reconstructed limb, but rather a bilateral group-level difference in frontal-plane hip movement control observed in the ACLR group. The smaller hip abduction angle indicates that frontal-plane hip motion was restricted during double-leg squatting, whereas the lower hip adduction moment reflects a reduced frontal-plane moment demand at the hip. Patients after ACLR may adopt a relatively conservative frontal-plane hip control strategy to maintain pelvic and lower-limb stability and accommodate the redistribution of bilateral loading during the squat. Previous research has shown that the mediolateral position of the hips during double-leg squatting is associated with asymmetries in bilateral vertical ground reaction forces and knee extensor moments [43], suggesting that frontal-plane hip control may contribute to the redistribution of lower-limb loading. Taken together, these findings indicate that clinical assessment should not focus solely on knee joint loading in the reconstructed limb. Trunk and pelvic position, bilateral frontal-plane hip control, and hip muscle strength should also be considered to provide a comprehensive evaluation of the patient’s overall lower-limb movement strategy.

The present findings support our second hypothesis. During double-leg squatting, patients 6 months after ACLR showed greater negative hip work and hip work contribution in the reconstructed limb than in both the contralateral limb and healthy controls. They also showed lower negative knee work and knee work contribution in the reconstructed limb than in both the contralateral limb and healthy controls. In addition, ankle work contribution was greater in the reconstructed limb than in the contralateral limb. No clear differences were found in joint work or work contribution between the contralateral limb and healthy controls. These findings are consistent with an intralimb compensatory pattern in the reconstructed limb during double-leg squatting. This pattern involves coordinated changes between the hip and knee joints and may reduce loading at the knee joint. Susan et al. [26] reported that, during double-leg squatting at 5 months after ACLR, individuals shifted extensor demand from the knee joint to the hip joint. This finding suggests the presence of intralimb compensation. Similar intralimb compensatory patterns have also been observed in vertical jumping [17] and forward lunging [44]. In these tasks, patients after ACLR reduced knee joint loading and increased hip joint loading in the reconstructed limb. These findings are partly consistent with the present results. The present study further highlights the important role of the ankle joint in the compensatory strategy. The increased ankle work contribution suggests that the distal joint also participates in energy redistribution. The greater ankle work contribution suggests that the ankle also participates in the observed inter-joint work distribution. However, because negative ankle work did not differ significantly and the present study was cross-sectional, the functional significance of this finding should be interpreted cautiously. Therefore, functional assessment and rehabilitation training should focus on multi-joint coordination and multidimensional functional indicators. This may help identify individual compensatory patterns more comprehensively.

The present findings have several clinical implications. Functional assessment after ACLR has commonly used high-load tasks, such as single-leg squatting and hopping, to identify lower-limb movement control deficits [45]. During these tasks, abnormal alignment in the frontal plane, such as dynamic knee valgus, is closely associated with the risk of ACL injury [46]. Previous studies have also suggested that the hip muscles play an important role in regulating frontal-plane knee stability [47]. However, the present study found that, during double-leg squatting, a low-load closed-chain task, the reconstructed limb of patients after ACLR was primarily characterized by sagittal-plane knee movement control deficits, whereas no evident differences in frontal-plane kinematics were observed. This pattern may reflect lower sagittal-plane knee loading capacity and knee extensor function at the time of testing. A rehabilitation program that focuses only on frontal-plane stability may not fully restore functional status. Greater emphasis should therefore be placed on restoring knee flexor and extensor strength, as well as sagittal-plane dynamic control. Progressive loaded squatting and symmetrical loading training may be considered in rehabilitation, although intervention studies are needed to determine whether these approaches modify the observed movement patterns. In addition, low-risk tasks such as double-leg squatting can also identify potential movement control deficits. These tasks have clinical value for functional assessment and may help guide stage-specific rehabilitation strategies.

This study has several limitations. First, the cross-sectional design limits temporal and causal interpretation of the findings. Because participants were assessed at only one postoperative time point, the present study cannot determine when the observed biomechanical patterns emerged or whether they persist over time. Second, although the present study identified predominantly sagittal-plane knee movement control alterations during double-leg squatting after ACLR, it did not investigate the underlying muscular mechanisms. Previous studies have shown that patients after ACLR often have reduced quadriceps strength and insufficient neuromuscular activation [48,49,50]. They may also show altered coordination patterns involving the hamstrings and gluteal muscles. These factors may affect knee loading capacity and multi-joint moment distribution. Future studies incorporating electromyography and musculoskeletal modelling are needed to further clarify the muscular mechanisms underlying these movement patterns. Finally, several potentially relevant clinical and surgical variables, including injury-to-surgery time, knee range of motion, test-day pain or effusion, residual laxity, detailed surgical characteristics, and rehabilitation exposure, were not systematically recorded. In addition, the ACLR cohort was clinically heterogeneous with respect to concomitant meniscal pathology and related procedures, and some participants underwent chondral debridement. These unmeasured and heterogeneous clinical factors may have influenced squat biomechanics; therefore, residual confounding cannot be excluded.

Future studies should validate these findings in larger multicenter cohorts with more homogeneous patient characteristics to improve the generalizability of the results. Larger samples would also allow subgroup comparisons according to factors such as age and physical activity level, which may influence movement control and inter-joint work redistribution after ACLR. As the present study was not designed or powered for subgroup analyses, these comparisons were not performed.

## 5. Conclusions

During double-leg squatting, patients 6 months after ACLR showed sagittal-plane knee movement control deficits in the reconstructed limb, accompanied by bilateral alterations in frontal-plane hip movement control. They also demonstrated a redistribution of joint work away from the knee toward the hip and ankle. Compared with the contralateral limb, knee work contribution was approximately 12.5 percentage points lower, whereas hip and ankle work contributions were approximately 11.5 and 0.9 percentage points greater, respectively. These cross-sectional findings characterize biomechanical differences present at approximately 6 months after ACLR and highlight the potential value of joint work distribution during a relatively low-load task for multi-joint functional assessment and identification of compensatory movement strategies; however, they do not establish when these patterns developed or whether they persist over time.

## Figures and Tables

**Table 1:** Comparison of Participant Characteristics.

	ACLR Group (*n* = 27)	Control Group (*n* = 23)	*p* Value
Age (years)	29.8 ± 7.2	27.6 ± 4.7	0.201
Sex, *n*	20M, 7F	20M, 3F	0.308
Body mass (kg)	74.7 ± 12.6	75.1 ± 10.6	0.927
Height (m)	1.8 ± 0.1	1.8 ± 0.1	0.667
Body mass index (kg/m^2^)	24.2 ± 3.0	24.1 ± 2.7	0.893
Preoperative Tegner activity level	7.0 (6.0, 7.0)	6.0 (6.0, 7.0)	0.053
6-month postoperative Tegner activity level	5.0 (4.0, 6.0)	NA	NA
Returned to sport at the time of testing, n (%)	0 (0%)	NA	NA
Hamstring concentric peak torque at 60°/s, contralateral limb (N·m/kg)	1.2 ± 0.3	NA	NA
Hamstring concentric peak torque at 60°/s, reconstructed limb (N·m/kg)	1.1 ± 0.3	NA	NA
Quadriceps concentric peak torque at 60°/s, contralateral limb (N·m/kg)	2.4 ± 0.6	NA	NA
Quadriceps concentric peak torque at 60°/s, reconstructed limb (N·m/kg)	1.8 ± 0.6	NA	NA
IKDC	77.3 ± 10.0	NA	NA
Lysholm	81.3 ± 11.4	NA	NA
Postoperative time, mo, mean ± SD	5.9 ± 0.7	NA	NA

F, female; M, male. NA, not applicable. IKDC, International Knee Documentation Subjective Knee questionnaire.

**Table 2:** Trunk and Lower-Limb Joint Angles at Peak Knee Extensor moment (°).

	ACLR		Healthy Controls		*p* (*η*^2^\*g*\*g_z_*)				
	Reconstructed Limb	Contralateral Limb	Non-Dominant Limb	Dominant Limb	Interaction Effect	Within-Group Main Effect		Between-Group Main Effect	
Trunk flexion	44.0 ± 11.4	45.1 ± 10.8	41.2 ± 9.3	40.9 ± 8.8	0.101 (0.055)	0.337 (0.019)		0.225 (0.031)	
Trunk lateral bending	−0.9 ± 2.6	−0.9 ± 2.6	−0.7 ± 1.5	−0.8 ± 1.5	0.583 (0.006)	0.798 (0.001)		0.789 (0.002)	
Trunk axial rotation	−0.5 ± 2.7	−0.5 ± 2.8	−0.4 ± 3.8	−0.2 ± 3.7	0.132 (0.047)	0.227 (0.030)		0.777 (0.002)	
Hip flexion	102.7 ± 11.8	104.2 ± 9.9	104.5 ± 8.1	103.6 ± 10.9	0.173 (0.038)	0.771 (0.002)		0.843 (0.001)	
Hip abduction	−15.9 ± 4.8	−19.2 ± 7.2	−21.1 ± 5.3	−21.6 ± 6.6	0.113 (0.052)	0.033 (0.091) *		0.014 (0.118) *	
Hip internal rotation	11.3 ± 8.7	6.9 ± 9.6	8.6 ± 8.7	7.8 ± 9.4	0.265 (0.026)	0.094 (0.057)		0.664 (0.004)	
Knee flexion	99.0 ± 8.5	103.6 ± 9.0	107.0 ± 10.1	106.6 ± 12.4	0.026 (0.099) *	0.003 (0.563) ^a^ *	0.824 (0.048) ^b^	0.004 (0.846) ^c^ *	0.321 (0.280) ^d^
Knee adduction	9.8 ± 6.5	6.9 ± 9.8	5.3 ± 6.1	5.3 ± 8.5	0.205 (0.033)	0.226 (0.030)		0.119 (0.050)	
Knee external rotation	−15.5 ± 10.1	−12.0 ± 6.5	−11.8 ± 10.9	−13.4 ± 9.1	0.055 (0.074)	0.449 (0.012)		0.606 (0.006)	
Ankle dorsiflexion	23.2 ± 6.7	27.4 ± 5.0	26.0 ± 5.4	27.2 ± 5.4	0.017 (0.112) *	<0.001 (0.836) ^a^ *	0.169 (0.357) ^b^	0.124 (0.437) ^c^	0.868 (0.047) ^d^
Ankle inversion	6.9 ± 5.9	4.8 ± 5.4	8.0 ± 5.4	7.4 ± 5.9	0.256 (0.027)	0.051 (0.077)		0.211 (0.032)	
Ankle internal rotation	21.6 ± 11.2	16.0 ± 9.4	22.7 ± 6.4	21.5 ± 7.8	0.107 (0.053)	0.015 (0.116) *		0.137 (0.045)	

Note: * indicates a significant interaction effect or main effect. ^a^ indicates comparisons between the reconstructed and contralateral limbs in individuals with Anterior cruciate ligament reconstruction (ACLR). ^b^ indicates comparisons between the non-dominant and dominant limbs in healthy controls. ^c^ indicates comparisons between the reconstructed limb in the ACLR group and the non-dominant limb in healthy controls. ^d^ indicates comparisons between the contralateral limb in the ACLR group and the dominant limb in healthy controls. Note: Positive values indicate flexion, adduction (or trunk lateral bending), and internal rotation (or trunk axial rotation) in the sagittal, frontal, and transverse planes, respectively.

**Table 3:** Lower-Limb Joint Moments at Peak Knee Extensor moment (Nm/kg).

	ACLR		Healthy Controls		*p* (*η*^2^\*g*\*g_z_*)				
	Reconstructed Limb	Contralateral Limb	Non-Dominant Limb	Dominant Limb	Interaction Effect	Within-Group Main Effect		Between-Group Main Effect	
Hip extension	−0.71 ± 0.26	−0.67 ± 0.23	−0.69 ± 0.33	−0.71 ± 0.36	0.241 (0.029)	0.742 (0.002)		0.895 (<0.001)	
Hip adduction	0.29 ± 0.11	0.31 ± 0.16	0.42 ± 0.18	0.45 ± 0.18	0.956 (<0.001)	0.125 (0.048)		0.001 (0.192) *	
Hip internal rotation	0.12 ± 0.14	0.07 ± 0.15	0.08 ± 0.16	0.07 ± 0.12	0.289 (0.023)	0.225 (0.031)		0.622 (0.005)	
Knee extension	−0.94 ± 0.20	−1.21 ± 0.21	−1.20 ± 0.36	−1.20 ± 0.28	<0.001 (0.300) *	<0.001 (1.220) ^a^ *	0.946 (0.014) ^b^	0.002 (0.909) ^c^ *	0.908 (0.032) ^d^
Knee abduction	−0.17 ± 0.14	−0.20 ± 0.25	−0.24 ± 0.17	−0.25 ± 0.28	0.792 (0.001)	0.442 (0.012)		0.262 (0.026)	
Knee external rotation	−0.14 ± 0.09	−0.12 ± 0.12	−0.20 ± 0.12	−0.23 ± 0.12	0.227 (0.030)	0.794 (0.001)		0.003 (0.164) *	
Ankle plantarfle-xion	−0.27 ± 0.16	−0.28 ± 0.19	−0.31 ± 0.13	−0.34 ± 0.17	0.633 (0.005)	0.239 (0.029)		0.219 (0.031)	
Ankle eversion	−0.04 ± 0.08	−0.01 ± 0.10	−0.04 ± 0.07	−0.01 ± 0.06	0.908 (<0.001)	0.092 (0.058)		0.916 (<0.001)	
Ankle internal (+)/external (−) rotation	0.02 ± 0.10	−0.01 ± 0.11	−0.04 ± 0.10	−0.05 ± 0.06	0.560 (0.007)	0.269 (0.025)		0.027 (0.097) *	

Note: * indicates a significant interaction effect or main effect. ^a^ indicates comparisons between the reconstructed and contralateral limbs in individuals with ACLR. ^b^ indicates comparisons between the non-dominant and dominant limbs in healthy controls. ^c^ indicates comparisons between the reconstructed limb in the ACLR group and the non-dominant limb in healthy controls. ^d^ indicates comparisons between the contralateral limb in the ACLR group and the dominant limb in healthy controls. Note: Positive values indicate flexion, adduction (or trunk lateral bending), and internal rotation (or trunk axial rotation) in the sagittal, frontal, and transverse planes, respectively.

**Table 4:** Peak Lower-Limb Joint Angles During Eccentric Phase of Squat (°).

	ACLR		Healthy Controls		*p* (*η*^2^\*g*\*g_z_*)				
	Reconstructed Limb	Contralateral Limb	Non-Dominant Limb	Dominant Limb	Interaction Effect	Within-Group Main Effect		Between-Group Main Effect	
Hip flexion	108.2 ± 10.4	107.7 ± 9.5	108.8 ± 9.5	107.6 ± 11.2	0.647 (0.004)	0.171 (0.039)		0.928 (<0.001)	
Hip abduction	−17.3 ± 5.0	−20.3 ± 7.1	−22.1 ± 5.1	−22.7 ± 6.6	0.181 (0.037)	0.035 (0.089) *		0.017 (0.112) *	
Hip internal rotation	14.3 ± 9.0	9.2 ± 9.5	10.9 ± 8.5	10.0 ± 9.5	0.206 (0.033)	0.067 (0.068)		0.533 (0.008)	
Knee flexion	106.8 ± 5.7	108.9 ± 7.1	112.4 ± 9.6	112.1 ± 10.1	0.017 (0.113) *	0.003 (0.603) ^a^ *	0.633 (0.093) ^b^	0.015 (0.707) ^c^ *	0.198 (0.365) ^d^
Knee adduction	11.1 ± 6.4	8.4 ± 9.4	7.4 ± 6.1	8.3 ± 6.6	0.086 (0.060)	0.407 (0.014)		0.284 (0.024)	
Knee external rotation	−27.6 ± 9.8	−23.6 ± 10.3	−24.1 ± 6.4	−27.5 ± 8.8	0.025 (0.100) *	0.070 (0.304) ^a^	0.159 (0.357) ^b^	0.154 (0.405) ^c^	0.168 (0.391) ^d^
Ankle dorsiflexion	24.2 ± 6.6	28.3 ± 4.7	26.6 ± 5.2	27.6 ± 5.0	0.014 (0.120) *	<0.001 (0.817) ^a^ *	0.244 (0.306) ^b^	0.169 (0.390) ^c^	0.647 (0.129) ^d^
Ankle inversion	8.3 ± 5.0	6.9 ± 4.5	9.1 ± 4.6	9.1 ± 5.5	0.260 (0.026)	0.287 (0.024)		0.231 (0.030)	
Ankle internal rotation	24.4 ± 10.8	20.5 ± 8.6	24.8 ± 6.2	25.3 ± 6.8	0.109 (0.053)	0.208 (0.033)		0.188 (0.036)	

Note: * indicates a significant interaction effect or main effect. ^a^ indicates comparisons between the reconstructed and contralateral limbs in individuals with ACLR. ^b^ indicates comparisons between the non-dominant and dominant limbs in healthy controls. ^c^ indicates comparisons between the reconstructed limb in the ACLR group and the non-dominant limb in healthy controls. ^d^ indicates comparisons between the contralateral limb in the ACLR group and the dominant limb in healthy controls. Note: Positive values indicate flexion, adduction (or trunk lateral bending), and internal rotation (or trunk axial rotation) in the sagittal, frontal, and transverse planes, respectively.

**Table 5:** Peak Lower-Limb Joint Moments During Eccentric Phase of Squat (Nm/kg).

	ACLR		Healthy Controls		*p* (*η*^2^\*g*\*g_z_*)				
	Reconstructed Limb	Contralateral Limb	Non-Dominant Limb	Dominant Limb	Interaction Effect	Within-Group Main Effect		Between-Group Main Effect	
Hip flexion	−1.06 ± 0.17	−1.00 ± 0.19	−0.99 ± 0.28	−1.01 ± 0.29	0.109 (0.053)	0.376 (0.016)		0.659 (0.004)	
Hip adduction	0.45 ± 0.11	0.48 ± 0.18	0.59 ± 0.18	0.63 ± 0.17	0.865 (0.001)	0.123 (0.049)		<0.001 (0.208) *	
Hip internal rotation	0.27 ± 0.14	0.25 ± 0.15	0.22 ± 0.17	0.22 ± 0.12	0.667 (0.004)	0.712 (0.003)		0.295 (0.023)	
Knee extension	−0.94 ± 0.20	−1.21 ± 0.21	−1.21 ± 0.36	−1.21 ± 0.29	<0.001 (0.314) *	<0.001 (1.216) ^a^ *	0.973 (0.007) ^b^	0.002 (0.912) ^c^ *	0.928 (0.025) ^d^
Knee abduction	−0.23 ± 0.12	−0.27 ± 0.24	−0.29 ± 0.16	−0.31 ± 0.26	0.756 (0.002)	0.376 (0.016)		0.300 (0.022)	
Knee external rotation	−0.24 ± 0.08	−0.22 ± 0.10	−0.29 ± 0.11	−0.32 ± 0.12	0.131 (0.047)	0.996 (<0.001)		0.003 (0.168) *	
Ankle plantarflexion	−0.36 ± 0.11	−0.35 ± 0.16	−0.37 ± 0.12	−0.39 ± 0.16	0.356 (0.018)	0.698 (0.003)		0.522 (0.009)	
Ankle eversion	−0.08 ± 0.07	−0.06 ± 0.09	−0.08 ± 0.06	−0.06 ± 0.06	0.849 (0.001)	0.167 (0.039)		0.748 (0.002)	
Ankle external rotation	−0.03 ± 0.06	−0.06 ± 0.07	−0.07 ± 0.04	−0.07 ± 0.04	0.153 (0.042)	0.209 (0.033)		0.017 (0.113) *	

Note: * indicates a significant interaction effect or main effect. ^a^ indicates comparisons between the reconstructed and contralateral limbs in individuals with ACLR. ^b^ indicates comparisons between the non-dominant and dominant limbs in healthy controls. ^c^ indicates comparisons between the reconstructed limb in the ACLR group and the non-dominant limb in healthy controls. ^d^ indicates comparisons between the contralateral limb in the ACLR group and the dominant limb in healthy controls. Note: Positive values indicate flexion, adduction (or trunk lateral bending), and internal rotation (or trunk axial rotation) in the sagittal, frontal, and transverse planes, respectively.

**Table 6:** Peak Ground Reaction Force During Eccentric Phase of Squat (N/kg).

	ACLR		Healthy Controls		*p* (*η*^2^\*g*\*g_z_*)				
	Reconstructed Limb	Contralateral Limb	Non-Dominant Limb	Dominant Limb	Interaction Effect	Within-Group Main Effect		Between-Group Main Effect	
Vertical ground reaction force	0.58 ± 0.07	0.64 ± 0.08	0.64 ± 0.14	0.66 ± 0.13	0.012 (0.125) *	<0.001 (0.838) ^a^ *	0.206 (0.344) ^b^	0.054 (0.552) ^c^	0.600 (0.147) ^d^

Note: * indicates a significant interaction effect or main effect. ^a^ indicates comparisons between the reconstructed and contralateral limbs in individuals with ACLR. ^b^ indicates comparisons between the non-dominant and dominant limbs in healthy controls. ^c^ indicates comparisons between the reconstructed limb in the ACLR group and the non-dominant limb in healthy controls. ^d^ indicates comparisons between the contralateral limb in the ACLR group and the dominant limb in healthy controls. Note: Positive values indicate flexion, adduction (or trunk lateral bending), and internal rotation (or trunk axial rotation) in the sagittal, frontal, and transverse planes, respectively.

**Table 7:** Negative Work and Joint Work Contribution of Lower Limbs During Eccentric Phase of Squat.

	ACLR		Healthy Controls		*p* (*η*^2^\*g*\*g_z_*)				
	Reconstructed Limb	Contralateral Limb	Non-Dominant Limb	Dominant Limb	Interaction Effect	Within-Group Main Effect		Between-Group Main Effect	
Hip negative work (J/kg)	−0.60 ± 0.16	−0.49 ± 0.17	−0.47 ± 0.15	−0.51 ± 0.17	<0.001 (0.272) *	<0.001 (0.880) ^a^ *	0.111 (0.310) ^b^	0.003 (0.874) ^c^ *	0.706 (0.106) ^d^
Knee negative work (J/kg)	−0.75 ± 0.18	−1.04 ± 0.22	−1.04 ± 0.28	−1.03 ± 0.27	<0.001 (0.356) *	<0.001 (1.501) ^a^ *	0.880 (0.028) ^b^	<0.001 (1.232) ^c^ *	0.936 (0.023) ^d^
Ankle negative work (J/kg)	−0.09 ± 0.04	−0.08 ± 0.05	−0.08 ± 0.04	−0.09 ± 0.04	0.111 (0.052)	0.824 (0.001)		0.538 (0.008)	
Hip work contribution (%)	42.19 ± 9.93	30.67 ± 9.95	29.87 ± 9.77	31.70 ± 10.47	<0.001 (0.451) *	<0.001 (1.391) ^a^ *	0.248 (0.260) ^b^	<0.001 (1.230) ^c^ *	0.724 (0.099) ^d^
Knee work contribution (%)	51.93 ± 9.47	64.41 ± 10.20	64.78 ± 10.32	62.65 ± 10.62	<0.001 (0.451) *	<0.001 (1.394) ^a^ *	0.219 (0.272) ^b^	<0.001 (1.282) ^c^ *	0.555 (0.166) ^d^
Ankle work contribution (%)	5.85 ± 2.87	4.93 ± 2.99	5.35 ± 2.21	5.65 ± 2.12	0.033 (0.091) *	0.018 (0.489) ^a^ *	0.463 (0.139) ^b^	0.495 (0.192) ^c^	0.335 (0.272) ^d^

Note: * indicates a significant interaction effect or main effect. ^a^ indicates comparisons between the reconstructed and contralateral limbs in individuals with ACLR. ^b^ indicates comparisons between the non-dominant and dominant limbs in healthy controls. ^c^ indicates comparisons between the reconstructed limb in the ACLR group and the non-dominant limb in healthy controls. ^d^ indicates comparisons between the contralateral limb in the ACLR group and the dominant limb in healthy controls. Note: Positive values indicate flexion, adduction (or trunk lateral bending), and internal rotation (or trunk axial rotation) in the sagittal, frontal, and transverse planes, respectively.

## Data Availability

The data presented in this study are available on request from the corresponding author. The data are not publicly available due to privacy and ethical restrictions.

## Abbreviations

The following abbreviations are used in this manuscript:

ACLR	Anterior Cruciate Ligament Reconstruction
ACL	Anterior Cruciate Ligament
IKDC	International Knee Documentation Subjective Knee questionnaire
GRF	Ground Reaction Force
